# Occurrence of Stroke and Reduced Ejection Fraction in Patients with Chagas Disease

**DOI:** 10.5935/abc.20180029

**Published:** 2018-03

**Authors:** Elieusa e Silva Sampaio, Márcia Maria Carneiro Oliveira, Roque Aras

**Affiliations:** Universidade Federal da Bahia, Salvador, BA - Brazil

**Keywords:** Chagas Disease, Chagas Cardiomyopathy, Stroke, Stroke Volume

**To the Editor**

Chagas disease (CD) is a well-defined risk factor for stroke.^1^ But the prognostic significance of stroke prevalence in left ventricular ejection fraction (LVEF) reduced compared to preserved LVEF in patients with heart failure and with CD, is still poorly known.^2^

There are studies that demonstrate the association of stroke with CD and reduced LVEF^3^ and there are studies that refute this association.^4^ In a cross-sectional study involving 85 chagasic patients with a mean age of 61.8 ± 9.3 years, 71.8% with heart failure and 96.5% of black race, patients were compared with LVEF ≤ 40% and LVEF > 40% to evaluate stroke occurrence in patients with CD and reduced LVEF. It was shown that LVEF ≤ 40% (OR 4.37: 1.65-11.63; p = 0.003) was an independent predictor for stroke compared to patients with preserved LVEF. There was also a high prevalence (50%) of CVA, which was obtained by cranial tomography, a number close to a cohort of 41.6% in the same town.^5^ There were no hemorrhagic strokes and there was also no significant relationship between fibrillation atrial and stroke, this data can be explained by the use of oral anticoagulants in these patients. In addition, 54.8% of silent vascular accidents were detected in patients who had no history of stroke. The high prevalence of stroke in this study with chagasic patients may have occurred because all patients were evaluated with cranial tomography, contrary to other studies, which generally use clinical and/or radiological findings as diagnostic criteria for stroke^1,4^ and do not usually evaluate silent cerebral infarction.^4^ Data suggest that reduced LVEF is associated with stroke, confirmed by cranial tomography and may be an independent predictor of embolic events in this population.
